# Characterization of wrapping and coating techniques for light-reflecting materials of LaBr_3_(Ce) scintillation detector

**DOI:** 10.1038/s41598-025-99401-1

**Published:** 2025-05-15

**Authors:** R. A. El-Tayebany

**Affiliations:** Nuclear and Radiological Safety Research Center, Egyptian Atomic Energy Authority, Cairo, Egypt

**Keywords:** MCNPX, LaBr_3_:Ce scintillators, Absolute efficiency, Wrapping & coating techniques, Energy science and technology, Engineering, Physics

## Abstract

Scintillation detectors need materials that reflect light in order to detect the incident photons or charged particles. It is necessary to attain an extremely high light accumulating efficiency. The purpose of this study is to investigate how coating and wrapping reflective materials which are employed to maximize light collection affect scintillator output. The MCNPX code was used for the simulation of the setup including LaBr_3_:Ce with the default reflector. The scintillators’ spectrum responses were evaluated with ^235^U, ^238^U, and ^137^Cs included. All the outcomes were discussed and explicated.

## Introduction

A scintillation element reflector is one of the most crucial components of a scintillation detector. In theory, a photodetector attached to the scintillator from one side should be able to catch all of the light released inside a material during a scintillation process. To guide as much of the produced light towards the photodetector as is practically possible, the scintillator’s reflecting coating is required. Therefore, the reflector’s characteristics have a big impact on how much light is gathered from the scintillator that is being used, which can dramatically enhance the detector’s overall performance. Teflon tape is frequently used in scintillation studies and is regarded as a common wrapping material^1,2^. It is obvious that the reflectors have an effect on raising the scintillation detectors’ light-collecting efficiency. Thus far, a number of research investigations have examined the characteristics of various reflectors. Determining the reflectivity was the objective of these studies. The degree of accuracy of the detector output is determined by a number of additional factors^3–5^. For instance, depending on whether the reflector is coated or wrapped around the scintillator, the detector’s response varies. In this instance, the matching refractive indices may have an impact on the detector output whenever there is an optical contact that exists between the scintillator and the reflector’s surface^6–8^. Commercial detectors usually use a metallic or plastic detecting cell that is reflective due to the usage of metallic foil. Over the past few years, there has been a rise in the usage of customized detectors. Using three photomultipliers, the triple-to-double coincidence ratio detectors, which scientists at National Metrology institutes first improved, are instances of these detectors. Furthermore, research organizations have created incredibly complex detectors intended to identify high-energy particles^9^. To improve performance, evaluation of the light transmission and reflecting material’s reflective properties is necessary, regardless of the detector’s intended use^10–12^. The most recent study has concentrated on the effects of reflector type, wrapping method, and related assembly method on rod-shaped plastic scintillator detectors’ output. In the studies, reflectors such as white paper, titanium dioxide, aluminum (Al)-foil tape, and aluminum-metalized Mylar (Al Mylar) were employed. The aforementioned reflectors’ scintillator outputs were examined with gamma sources ^60^Co and ^137^Cs included^8^. In the research of Kensington N. Vincent et al., they concentrated on developing extremely low-energy sensitivity detectors, which are necessary for detecting small changes in closely spaced energy peaks in the hunt for Dark Matter. On the innermost layer of the detector, a different wrapping material will be employed to improve the potential of CsI(Tl) scintillator detectors to do so. The material used for this covering is called 3MTM Enhanced Specular Reflector (ESR). Two types of measurements using ^60^Co and ^241^Am sources are performed to test this notion. The outcomes demonstrated that our detector can more easily collect photons produced by low-energy interactions because of the high photon reflectivity capabilities of the ESR layer^13^. In detector locations where the light collection is insufficient, D. Gallacher et al. employed a different technique with liquid-argon-based dark matter detectors that allows the separation of alpha-decays from nuclear-recoil-like events. The characteristics and production process of pyrene-doped polystyrene, a polymeric film that shifts wavelengths and was developed for the DEAP-3600 detector, are presented. By placing this film on the detector neck—a place outside the active volume with low scintillation light collecting efficiency—alpha particles’ observable energy and scintillation pulse shape are modified. They predict that the coating is expected to provide a 10^5^ suppression factor versus these events based on pulse shape discrimination^14^. The study examines light extraction techniques for LYSO and BGO, two of the most widely used inorganic scintillators. Francesco Gramuglia et al. provide new techniques that use metal coatings, modified Photonic Crystal (PhC) structures, and Distributed Bragg Reflectors (DBRs) and explain and compare them with conventional techniques. They were able to achieve an upper limit of around 21% light gain on energy resolution and 41% on light extraction for BGO, which has increased in acceptance recently because of its quick Cherenkov constituent and reduced expense^15^. To examine the attenuation properties of scintillation photons from Gd_3_A_l2_Ga_3_O_12_ (Ce: GAGG) scintillators doped with Ce, more study was conducted on samples of different sizes. The experiment’s narrow Ce: GAGG scintillator had an attenuation coefficient of 0.010 ± 0.001 mm ^− 1^ by using the reflector wrapping. While in the beginning, the Ce: GAGGs attenuation coefficient was found to be 0.00155 ± 0.00006 mm^− 1^. Therefore, the geometrical attenuation had a significant effect on the number of photons detected in this arrangement, although the self-absorption effect only contributed 10% of the overall attenuation^16^. The development of light-reflecting materials for pixelated scintillator detectors was investigated by Petr S. Sokolov et al. The reflecting surfaces for pixels size hBN between 0.8 and 3.2 mm were made using an inexpensive DLP 3D (Digital Light Processing) printing method. It is a 3D printing technology used to rapidly produce photopolymer parts. The reflectors made of the novel composite, which contained TiO_2_, were utilized. It was discovered that TiO_2_ performed better than BaSO_4_, Hexagonal Boron Nitride (hBN), and cubic zirconia pigments. The reflectors are created at a rate of about 1 centimeter every hour, and the wrapping procedure is made easier by the capacity to make many sections simultaneously. It was found that a scintillator could gather more light if the manufactured reflectors had a regular pattern^17^. An increase in the number of optical photons (due to higher scintillation efficiency or better light collection) improves the ability to accurately measure the energy of the gamma photons. In this work, we used two types of techniques that are used in scintillation detectors for reflecting material manufacturing. The two types are coating and wrapping techniques which reflect mainly on scintillation detector performance. Here, we are concerned with the effect of wrapping and coating techniques on the output of LaBr3:Ce scintillator detectors and evaluated the manufacturing technique itself on the wavelength of the optical photons produced as a result of incident gamma radiation on the reflector material and their interaction with the scintillator medium.

## Wrapping and coating techniques

**Fig. 1:**
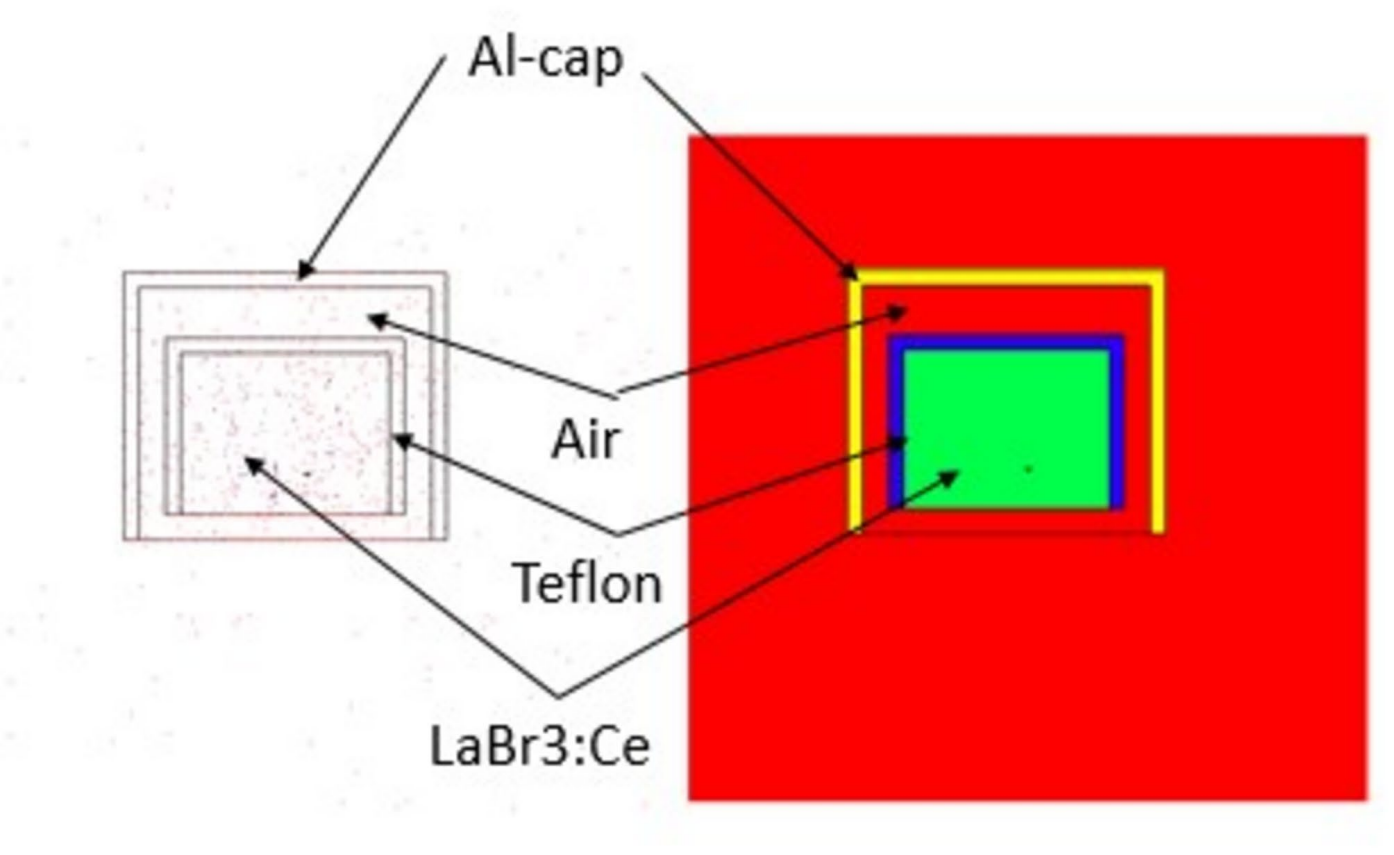
The simulated LaBr3:Ce detector by MCNPX in 2-D.

The two primary elements that determine a scintillation detector’s quality are its scintillator substance and its detector wrapper. High detection efficiency is provided by the bars’ dimensions and geometric configurations. Similar arrangements’ primary issue is thin tubes’ ineffective light transfer over long distances. Only materials with high reflectivity and specular reflectance can provide efficient light transport. To detect the tiny signals, a very good light collection is required, thus choosing the right wrapping material and making sure the foil fits precisely on the plastic were crucial requirements. There are several potential options, including plastic scintillator wrapping materials. Teflon, plastic, aluminum foils, 3 M radiant mirror films, white diffuse paint or tape, etc. are some of the materials that are in demand. Their high reflection rate is one of their key characteristics. These materials also need to be flexible within predetermined bounds and mechanically stable^18^. The wrapping is a thin layer that goes over the scintillator but the coating, on the other hand, actually bonds with the scintillator surface. Controlling the concentration and volume of paint applied allowed for the achievement of the coating thickness^19^. The material card found in the MCNP (Monte Carlo N-Particle) input file was used to determine the coating thickness. In reality, the coating thickness was obtained by adjusting the paint’s concentration as well as its volume, which was accomplished by inserting the material card into the MCNP simulation code’s input file. Once the reflector has been positioned around the crystal, the detector is often mostly covered with a black plastic tape. In doing so, the package design is strengthened and external light is prevented from entering the detector. The reflector’s optical characteristics may be impacted by this type of wrapping, which could alter the anticipated detector efficiency. There is a higher likelihood of optical photons in rod plastics self-absorbing and escaping because they have to travel a greater mean path to reach the PMT. The simulated LaBr_3_:Ce crystal is in a hermetically sealed aluminum housing cylinder 1.5 × 1.5 in. with a default reflector (Teflon) thickness 0.3 cm. The simulated scintillation detector is shown as in Fig. 1 using MCNPX. The number of optical photons produced per 1 MeV of deposited energy is approximately 63,000 photons. For a LaBr₃:Ce scintillator and Teflon reflector, the refractive indices for LaBr₃:Ce scintillator is typically around 1.9 to 2.0 and the Teflon has a refractive index of approximately 1.35. Teflon is known for its high diffuse reflectance, which can exceed 95% in the visible spectrum. This makes it an excellent material for reflecting scintillation light back into the detector. It maintains high reflectivity across a broad spectral range, from UV (ultraviolet) to IR (infrared), which is particularly useful in scintillation applications. It is highly resistant to UV radiation, which contributes to its stability and durability when used in scintillation detectors, where UV photons are often produced. These properties make Teflon a preferred choice for lining the inner surfaces of scintillation detectors to enhance light collection efficiency^20^.

## Results and discussion

**Fig. 2:**
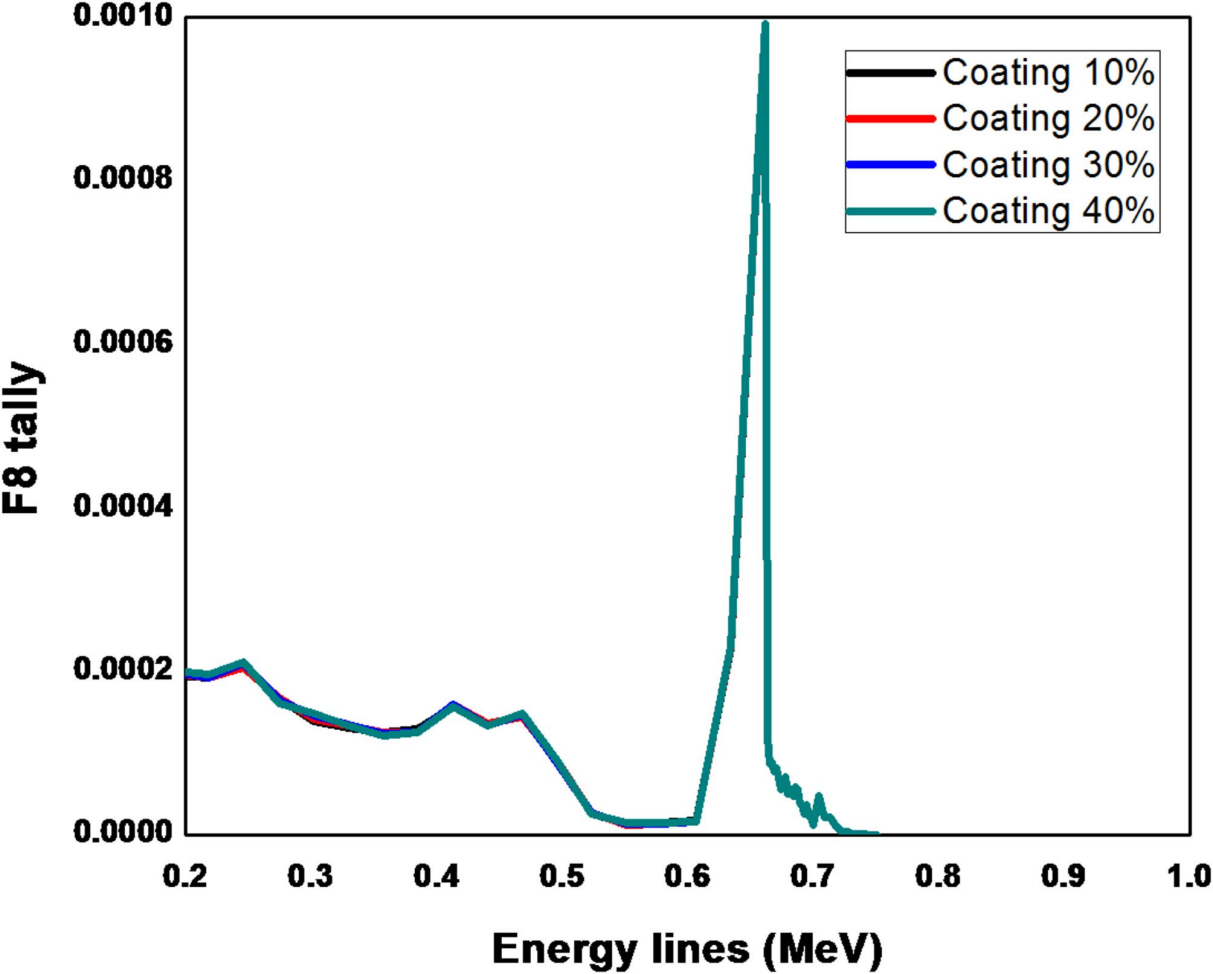
Pulse height distribution for ^137^Cs at 661.7 keV using Teflon coating reflector increased from 10 to 40% of its actual thickness.

**Fig. 3:**
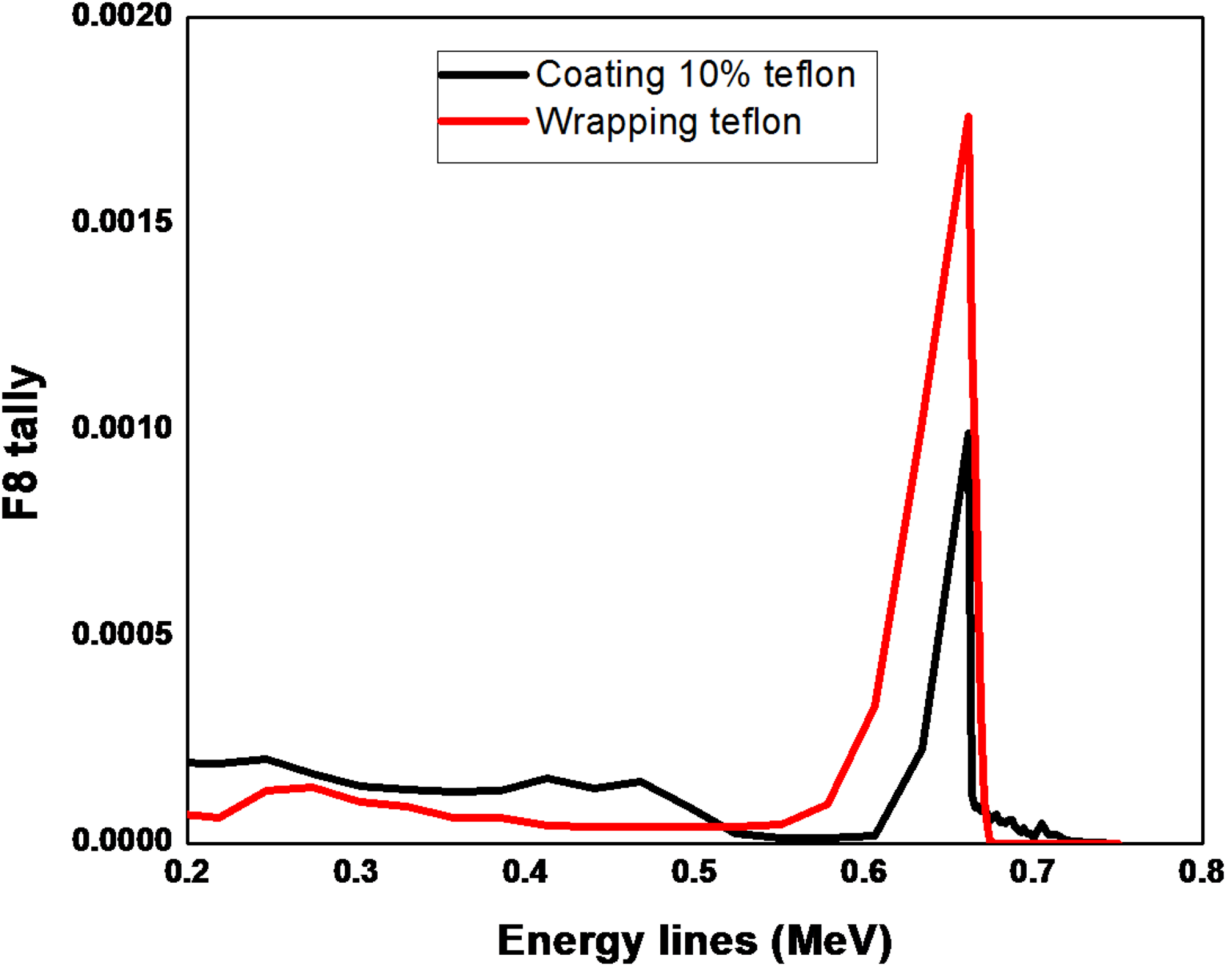
Pulse height distribution for ^137^Cs at 661.7 keV using Teflon reflector (coating (10%) & wrapping).

The results in Fig. 2 show the energy deposition distribution for ^137^Cs at energy line 661.7 keV. The coating thickness of the Teflon reflector is increased from 10 to 40% of its actual thickness. The F8 tally card in MCNP, often referred to as pulse-height tally, is used to calculate the energy deposited in a specific region or cell. This tally is particularly useful for simulating the response of a detector or for evaluating energy deposition in a volume. The F8 tally provides detailed information on the energy distribution of particles interacting within the defined region, allowing for an analysis of the radiation spectrum and the corresponding energy deposition. The results are typically presented as the incident photon energy versus the photon counts. The simulating spectrum obtained from MCNPX code illustrates that there is no difference in the peak shape but there is a slightly difference in the background from 200 to 500 keV which does not have a significant impact on the photopeak. On the other hand in Fig. 3, there is a great difference between simulating the wrapping reflector technique and the coating technique. The reflecting materials and their manufacturing techniques aim to improve the measurement and resolution of the gamma spectrum by enhancing the detection of optical photons. This effect is reflected on the photopeak intensity at 661.7 keV and the Compton region and background at (200–500) keV as well. The outcomes demonstrated that, in the 200–700 keV range, the wrapping technique outperforms the coating technique. In this area, the background became less and the photopeak count grew. The explaination for this relates to the increase in optical photon energy and the probability of traveling across the coating reflector surface, which leads to the escape of a large number of optical photons, potentially reaching half of the total deposited photons. Therefore, the wrapping reflector is more ideal than the coating one in this spectrum region. Another explaination, mentioned in Ref.^8^, refers to establishing the optical interface connecting the reflector and scintillator that raises the possibility that the optical photons will escape through refraction.

**Fig. 4:**
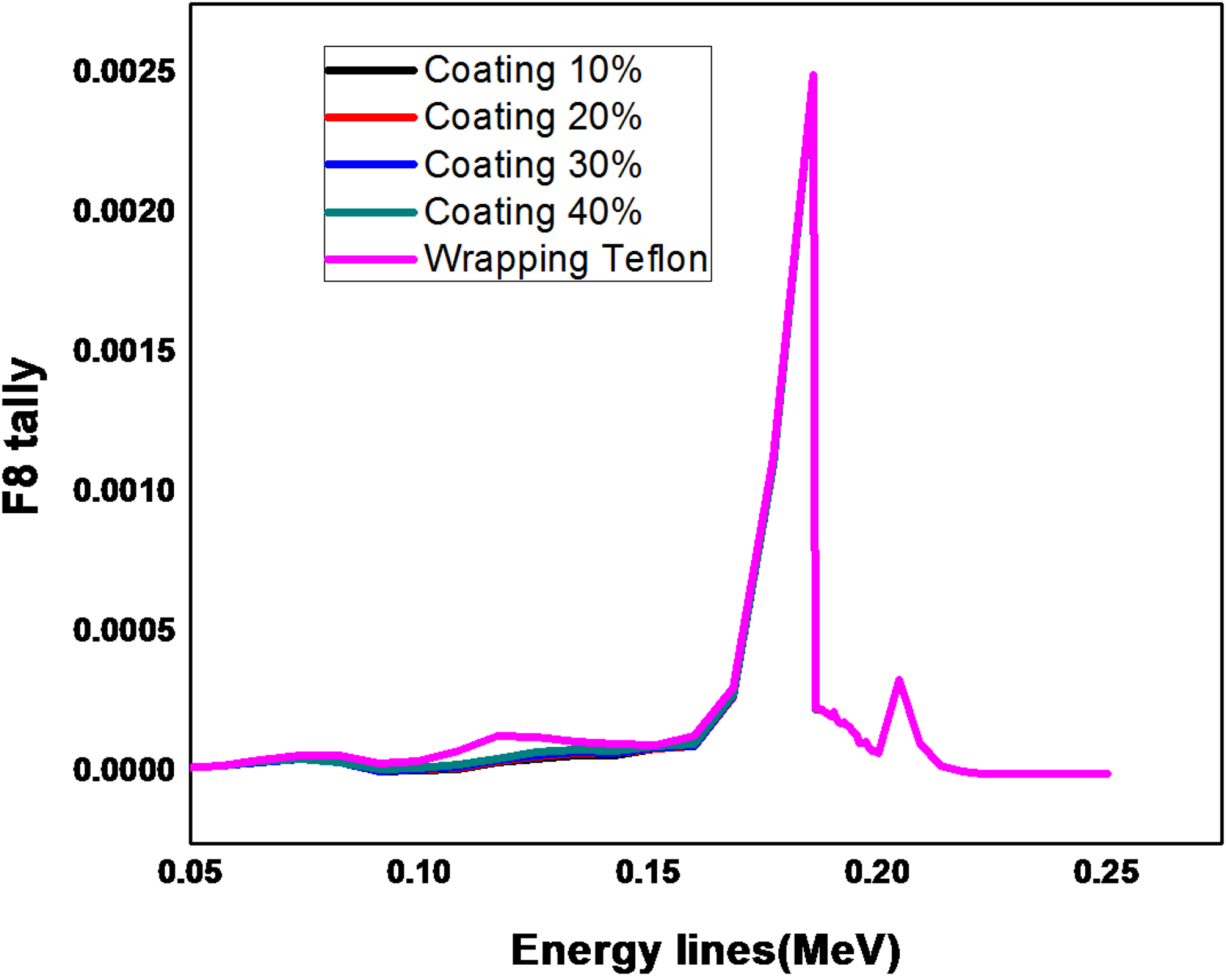
Pulse height distribution for ^235^U at 185.7 keV using Teflon reflector (coating (10-40)% & wrapping).

**Fig. 5:**
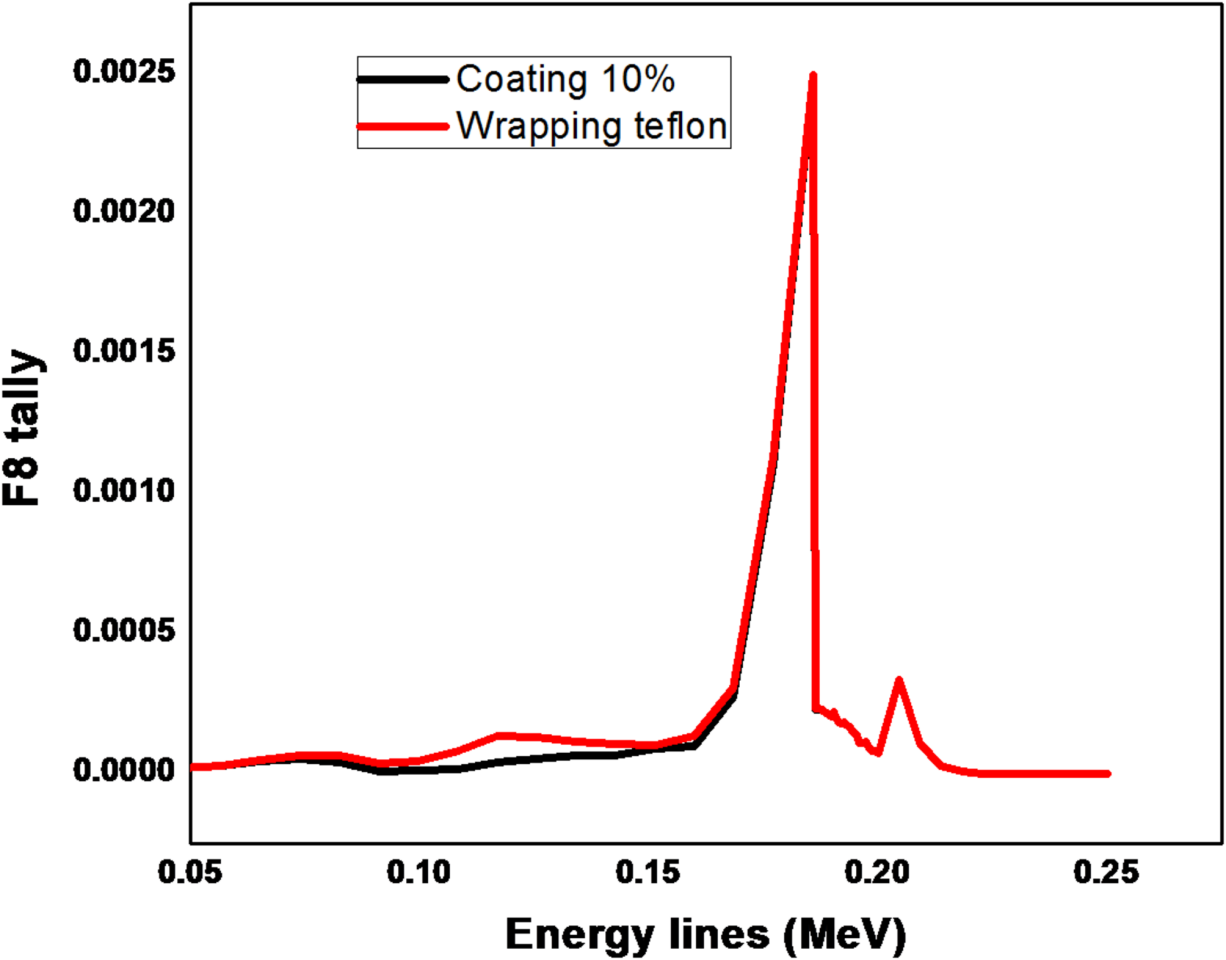
Pulse height distribution for ^235^ U at 185.7 keV using Teflon reflector (coating (10%) & wrapping).

By using the ^235^U at energy line 185.7 keV as in Fig. 4, the results of F8 tally shows that the area under the peak is identical in all cases by using the coating and wrapping Teflon reflector. However, the energy region from 50 to 150 keV has a very small difference which does not affect the peak at 185.7 keV and that is clear in Fig. 5 which describes the results of one case of using a coating reflector compared with results of wrapping reflector.

**Fig. 6:**
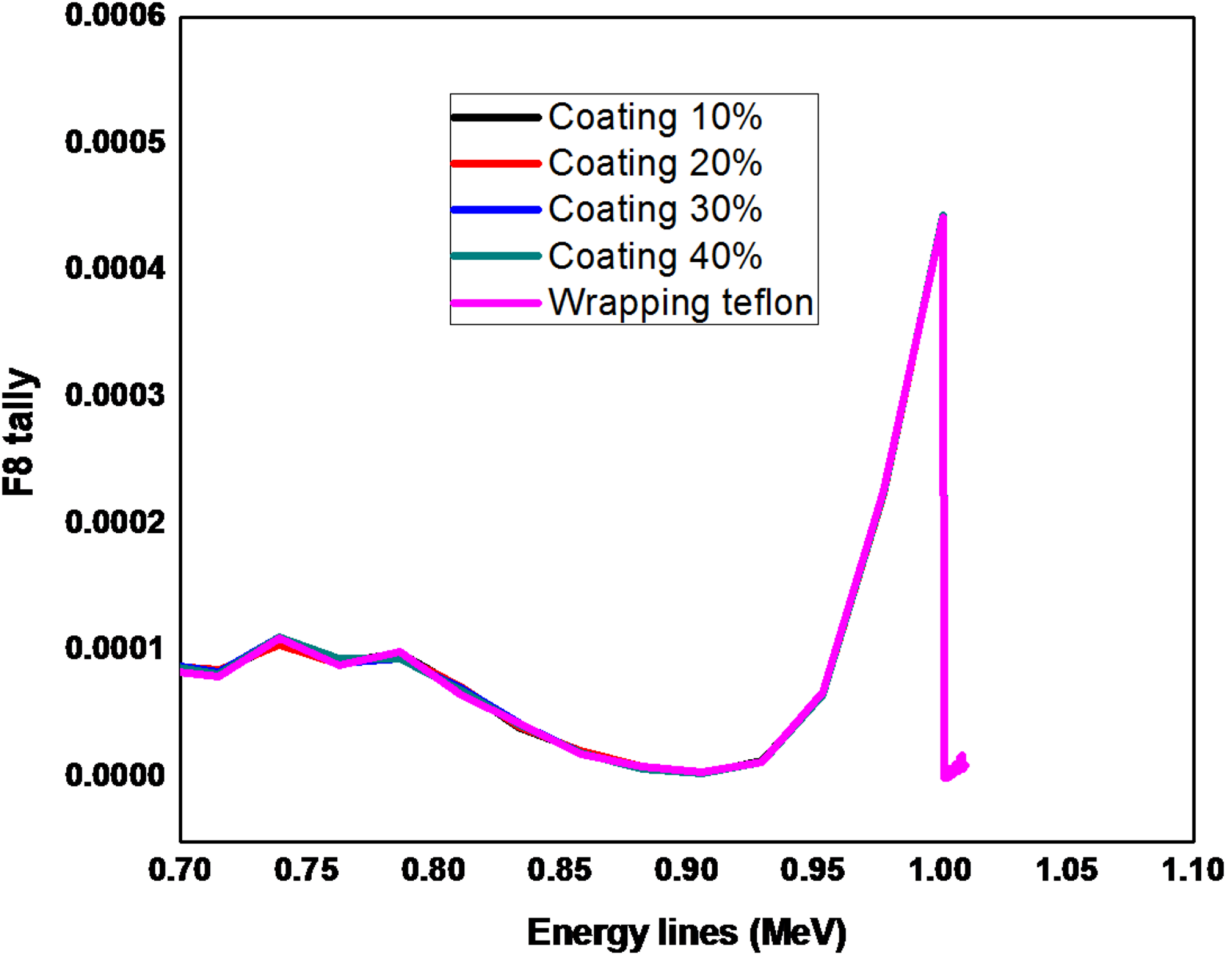
Pulse height distribution for ^238^U at 1001.2 keV using Teflon reflector (coating (10–40)% & wrapping).

At a high energy range above 700 keV, the energy line of ^238^U at 1001.2 keV is used in the simulation as presented in Fig. 6. The results show that there is a very slight difference in the counts in all cases. The reason why the number of escaping photons is nearly the same for all the reflector techniques is that, as the photon energy increases to an extreme level, the reflector becomes ineffective in collecting them.

## Conclusion

By applying the MCNPX code for the simulation of the scintillation detector using the default reflector (Teflon) and using different values of energy ranges, the wrapping reflector proved to provide the best results in the region 200–700 keV. The explanation for that refers to the increase in photon energy and the higher probability of traveling across the coating reflector surface, which leads to the escape of a large number of photons, potentially reaching half of the total deposited photons. Therefore, the wrapping reflector is more ideal than the coating one in this spectrum region. Rather than directly coating the reflector on the scintillator surface, the detector can be made more efficient by simply wrapping the reflector around the scintillator.

## Data Availability

Data is provided within the manuscript file.
